# Differential Functional Responses of Neutrophil Subsets in Severe COVID-19 Patients

**DOI:** 10.3389/fimmu.2022.879686

**Published:** 2022-05-31

**Authors:** Kenneth R. McLeish, Rejeena Shrestha, Aruna Vashishta, Madhavi J. Rane, Michelle T. Barati, Michael E. Brier, Mario Gutierrez Lau, Xiaoling Hu, Oscar Chen, Caitlin R. Wessel, Travis Spalding, Sarah E. Bush, Kenechi Ijemere, C. Danielle Hopkins, Elizabeth A. Cooke, Shweta Tandon, Terri Manning, Silvia M. Uriarte, Jiapeng Huang, Jun Yan

**Affiliations:** ^1^Division of Nephrology and Hypertension, Department of Medicine, University of Louisville, KY, United States; ^2^Department of Microbiology and Immunology, University of Louisville, KY, United States; ^3^Department of Oral Immunology and Infectious Diseases, School of Dentistry, University of Louisville, KY, United States; ^4^Division of Immunotherapy, The Hiram C. Polk, Jr., MD Department of Surgery, Immuno-Oncology Program, Brown Cancer Center, University of Louisville, Louisville, KY, United States; ^5^Department of Anesthesiology and Perioperative Medicine, University of Louisville, KY, United States

**Keywords:** neutrophil, COVID-19, low density neutrophil, respiratory burst, exocytosis, NET formation, T cell proliferation

## Abstract

Neutrophils play a significant role in determining disease severity following SARS-CoV-2 infection. Gene and protein expression defines several neutrophil clusters in COVID-19, including the emergence of low density neutrophils (LDN) that are associated with severe disease. The functional capabilities of these neutrophil clusters and correlation with gene and protein expression are unknown. To define host defense and immunosuppressive functions of normal density neutrophils (NDN) and LDN from COVID-19 patients, we recruited 64 patients with severe COVID-19 and 26 healthy donors (HD). Phagocytosis, respiratory burst activity, degranulation, neutrophil extracellular trap (NET) formation, and T-cell suppression in those neutrophil subsets were measured. NDN from severe/critical COVID-19 patients showed evidence of priming with enhanced phagocytosis, respiratory burst activity, and degranulation of secretory vesicles and gelatinase and specific granules, while NET formation was similar to HD NDN. COVID LDN response was impaired except for enhanced NET formation. A subset of COVID LDN with intermediate CD16 expression (CD16^Int^ LDN) promoted T cell proliferation to a level similar to HD NDN, while COVID NDN and the CD16^Hi^ LDN failed to stimulate T-cell activation. All 3 COVID-19 neutrophil populations suppressed stimulation of IFN-γ production, compared to HD NDN. We conclude that NDN and LDN from COVID-19 patients possess complementary functional capabilities that may act cooperatively to determine disease severity. We predict that global neutrophil responses that induce COVID-19 ARDS will vary depending on the proportion of neutrophil subsets.

## Introduction

Acute respiratory distress syndrome (ARDS) is a life-threatening disorder induced by inflammatory injury to the lungs initiated by local (eg. pneumonia) or systemic (eg. sepsis, trauma) disorders (1). Neutrophil recruitment and activation, leading to the release of reactive oxygen species (ROS), degranulation, and neutrophil extracellular trap (NET) formation, are critical components of ARDS pathophysiology (1, 2). Among patients hospitalized with severe acute respiratory syndrome coronavirus 2 (SARS-CoV-2)-induced coronavirus disease 2019 (COVID-19), approximately 1/3 develop ARDS, making COVID-19 the current leading cause of ARDS (3–6). Despite some potential differences in pathophysiology, accumulating evidence indicates that neutrophils also play a critical role in the development of COVID-19 ARDS. Lung specimens taken at autopsy show a marked infiltration of neutrophils and the presence of NETs. In addition, neutrophils are the predominant immune cells in bronchoalveolar lavage fluid (BALF) from COVID-19 patients with ARDS, and neutrophil serine proteases are present in BALF (7–22).

Patients with severe COVID-19 exhibit marked alterations in innate and adaptive immunity, including changes in neutrophil abundance, phenotype, and function (22–26). Neutrophil characterization by transcriptional and proteomic analysis in COVID-19 indicates significant heterogeneity among mature neutrophils and emergence of a large number of low density neutrophils (LDN) with multiple phenotypes (16, 24, 25, 27–30). The functional capability of these various neutrophil populations has been inferred from gene or protein expression profiles (22–26), however, previous direct functional studies either did not evaluate the various neutrophil populations or disparate results were reported (12–15, 24, 28–37). Thus, the functional heterogeneity among the various neutrophil populations remains to be defined. The emergence of LDN in the circulation and lungs (16, 24, 25, 27, 37), particularly in severe and critical COVID-19, indicates that defining the spectrum of functional alterations of different neutrophil populations is important to understanding the contribution of neutrophils to COVID-19 ARDS pathophysiology. To address this gap in knowledge, the current study compares the functional responses of LDN from COVID-19 patients (COVID LDN) with the responses of normal density neutrophils from COVID-19 patients (COVID NDN) and of NDN from healthy donors (HD NDN). Our results indicate that functional heterogeneity correlates with phenotypic heterogeneity and suggest the possibility that different neutrophil populations cooperate to induce ARDS in COVID-19.

## Results

### Characteristics of COVID-19 Patients

Between November, 2020 and December, 2021, we recruited 64 patients who tested positive for SARS-CoV-2 by nasopharyngeal swab and were assessed to have severe or critical COVID-19, as defined by the WHO criteria (38). The study participant demographics and summary of clinical information are shown in **Table 1**. The mean age was 59.8 ± 14.6 years. ICU admission was required in 59 of the 64 patients, 57 required mechanical ventilatory support, and 6 were supported with ECMO. The PaO2:FiO2 was below 100 in 25 patients, between 100 and 200 in 29, between 200 and 300 in 7, and above 300 in 3 who required mechanical ventilation. Comorbidities known to be risk factors for severe COVID-19 were present in 56 of the 64 patients, the most common of which were hypertension (n=39, 61%) and obesity (n=42, 66%). Elevated neutrophil to lymphocyte ratio (NLR), D-dimer, ferritin, and C-reactive protein were common, as previously reported (16, 39–42). The mean/median hospital day on which neutrophils used in this study were obtained was 8.5/6.5. Treatment with corticosteroids, remdesivir, and monoclonal antibodies or convalescent plasma occurred in 59 (92%), 48 (75%), and 29 (45%) patients, respectively. Outcome was poor, as 32 (50%) patients died during hospitalization and 19 (30%) were discharged to a long-term care or rehabilitation facility. A total of 26 healthy donors (HD) were recruited from the students, staff, and faculty at the University of Louisville. Demographic data was not recorded for HD, but the pool of subjects eligible for recruitment was younger than the patient population.

**Table 1:** Clinical characteristics and demographics.

n	64
**Median Age (range)**	59 (24-96)
**Male (%)**	34 (53%)
**Race (%)**	
White or Caucasian	48 (75%)
Black or African American	13 (20%)
Hispanic	1 (2%)
Not recorded	2 (3%)
**Weight (Kg)**	99.8 (41-190)
**BMI** median (range)	35.6 (17.2-66.1)
**Comorbidity**	
Obesity, patient number (%)	42 (66%)
Hypertension, patient number (%)	39 (61%)
Diabetes Mellitus, patient number (%)	26 (41%)
Coronary artery disease/arrhymia/CHF, patient number (%)	20 (31%)
Underlying lung disease, patient number (%)	21 (33%)
Chronic kidney disease, patient number (%)	7 (11%)
Hyperlipidemia, patient number (%)	14 (22%)
**Hospital Day Neutrophils Obtained** mean/median (range)	8.5/6.5 (1-36)
**Laboratory Values**	
WBC/Neutrophil (4.1-10.8 x10^3^/μL) mean/median	13.6/11.7
NLR mean/median	20.5/12.5
d-dimer (0.19-0.74 μg/ml FEU)	423, range 0.37-15,107 (n=40)
Ferritin (7-350 ng/ml)	711, range 71-7500 (n=34)
C-reactive protein (<3.0 mg/L)	177, range 2.6-380 (n=30)
**Hospital Course**	
APACHE	21, range 6-38
ICU, patient number (%)	59 (92%)
Ventilator, patient number (%)	57 (89%)
ECMO, patient number (%)	6 (9%)
Dialysis, patient number (%)	16 (25%)
Died, patient number (%)	32 (50%)
Discharge Home, patient number (%)	9 (14%)
Discharge to LTC, patient number (%)	22 (34%)
**Treatment**	
Remdesivir, patient number (%)	48 (75%)
Corticosteroids, patient number (%)	59 (92%)
Monoclonal antibody or Convalescent Plasma, patient number (%)	29 (45%)

### Phagocytosis and Respiratory Burst

**Figure 1:**
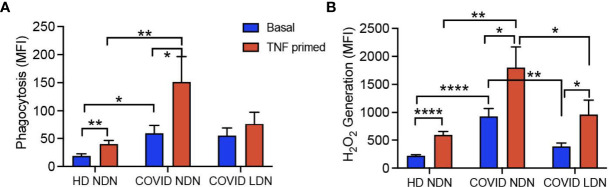
Phagocytosis and respiratory burst are enhanced in COVID-19 NDN, but not LDN. Results show basal and *in vitro* TNFα-primed (2 ng/ml) phagocytosis of opsonized, propidium iodide-labeled S. aureus **(A)** and subsequent H_2_O_2_ production **(B)** in COVID-19 NDN (n=11), LDN from COVID-19 (COVID LDN) (n=11), and NDN from healthy donors (HD NDN) (n=6). Results are expressed as mean ± SEM of mean fluorescence intensity (MFI) of each marker, determined by flow cytometry. *p < 0.05, **p < 0.01, ****p < 0.001.

Neutrophil phagocytosis and respiratory burst activity are critical for host defense function (43), and release of ROS is suggested as a contributing mechanism of lung injury leading to ARDS in COVID-19 (44, 45). Previous reports presented contradictory results for both neutrophil phagocytosis and respiratory burst activity (8, 25), and the response of LDN has not been reported. To address this gap in knowledge, we measured bacterial phagocytosis and phagocytic stimulation of H_2_O_2_ production in LDN and NDN neutrophils isolated from 11 COVID-19 patients and compared their responses to those of NDN from 6 HD. **Figure 1A** shows phagocytosis before and after *in vitro* priming with TNFα. Phagocytosis by unprimed cells was significantly greater in COVID NDN, compared to HD NDN. COVID LDN showed a level of phagocytosis intermediate between the other 2 groups and was not significantly different from either. TNFα priming significantly enhanced phagocytosis of both HD NDN and COVID NDN, with a significantly greater response by COVID NDN. Phagocytosis by COVID LDN did not increase with *in vitro* priming. **Figure 1B** shows phagocytosis-stimulated H_2_O_2_ production in the three groups of neutrophils. H_2_O_2_ production by COVID NDN, but not COVID LDN, was significantly greater than that by HD NDN. TNFα priming significantly enhanced H_2_O_2_ production to an equivalent degree in all three groups, with COVID NDN production remaining significantly greater than HD NDN or COVID LDN. We interpret these results to indicate that NDN from severe COVID-19 patients are primed for enhanced phagocytosis and respiratory burst. Despite existing in the same milieu, LDN are not primed for either enhanced phagocytosis or respiratory burst activity. Respiratory burst activity by COVID LDN was enhanced to the same degree as HD NDN in response to *in vitro* priming, indicating LDN are responsive to some priming agents.

### Degranulation

**Figure 2:**
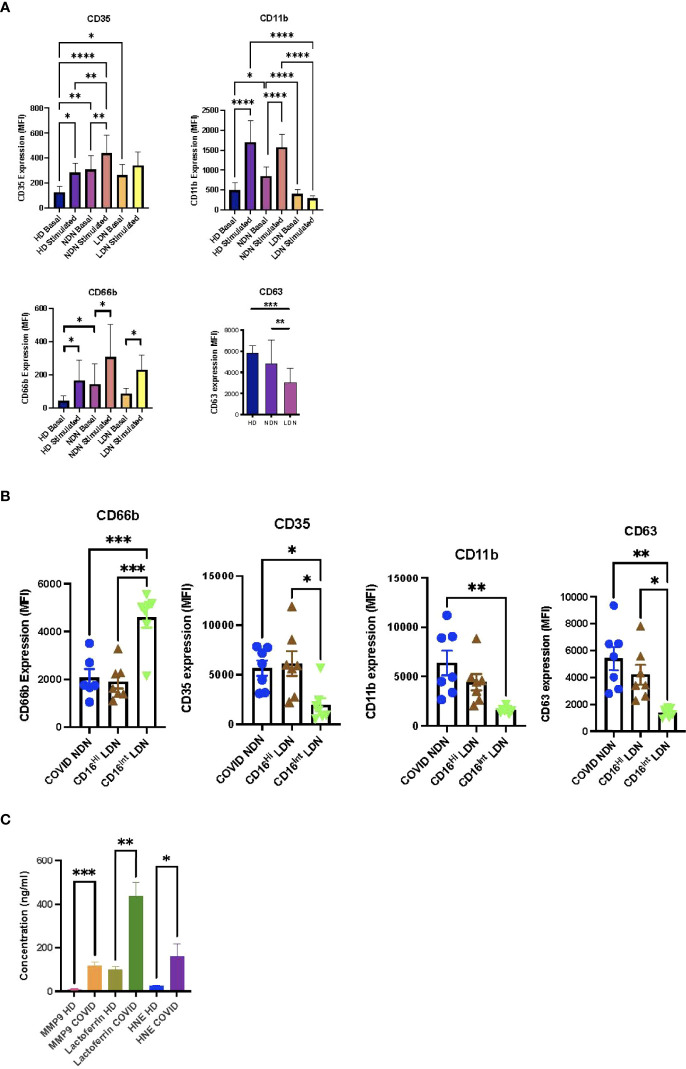
Differential degranulation by neutrophil subsets from COVID-19 patients. **(A)** Basal and stimulated (TNFα 2 ng/ml + fMLF 300 nM) expression of markers for secretory vesicles (CD35), gelatinase granules (CD11b), specific granules (CD66b), and azurophilic granules (CD63) on COVID NDN (n=11-15), COVID LDN (n=11-15), and HD NDN (n=8-9). Results are expressed as mean ± SEM of mean fluorescent intensity (MFI). *p < 0.05, **p < 0.01, ***p < 0.001, ****p < 0.0001. **(B)** Comparison of basal expression of CD35, CD11b, CD66b, and CD63 on COVID NDN, CD16^Hi^ LDN, and CD16^Int^ LDN (n=7). Results are expressed as mean ± SEM of MFI. *p < 0.05, **p < 0.01, ***p < 0.001. **(C)** Plasma levels of MMP9, lactoferrin, and human neutrophil elastase in plasma from HD (n=10) and COVID-19 (n=10-25). Results expressed as mean + SEM in ng/ml. *p < 0.05, **p < 0.01, ***p < 0.001.

Previous neutrophil degranulation studies using expression of plasma membrane markers showed enhanced azurophilic granule exocytosis, measured by CD63, in COVID-19 (28, 29, 31). Contradictory results were reported for exocytosis of specific (CD66b) and gelatinase (CD11b) granules (9, 28, 29, 31, 32, 34, 35, 46). To evaluate degranulation of neutrophil populations from COVID-19 patients, we compared expression of plasma membrane markers of the 4 neutrophil granule subsets, CD35 (secretory vesicles), CD11b (gelatinase granules), CD66b (specific granules), and CD63 (azurophilic granules), among HD NDN, COVID NDN, and COVID LDN before and after stimulation (**Figure 2A**). Basal CD35 expression was significantly greater in COVID NDN and COVID LDN than in HD NDN, indicating that similar secretory vesicle degranulation occurred *in vivo* in LDN and NDN in COVID-19 patients. Stimulation with TNFα and fMLF increased CD35 expression in HD NDN and COVID NDN, but not COVID LDN, suggesting LDN underwent maximal release of secretory vesicles *in vivo*. Basal CD11b expression by COVID NDN was significantly greater than expression by HD NDN or COVID LDN. Additionally, TNFα and fMLF stimulation increased CD11b expression in HD NDN and COVID NDN, but not in COVID LDN. Basal CD66b expression was significantly greater in COVID NDN, but not COVID LDN, than in HD NDN. *In vitro* stimulation resulted in a significant increase in CD66b in all 3 groups of cells. CD63 expression did not differ between HD NDN and COVID NDN. CD63 expression by COVID LDN was significantly lower than either group of NDN. The response of CD63 to *in vitro* stimulation was not tested, as pharmacologic disruption of the actin cytoskeleton is required (47). Thus, markers for the different neutrophil granule subsets showed distinct patterns of expression, suggesting differences in granule marker expression or in granule mobilization both among granule subsets and between NDN and LDN in COVID-19. We interpret these results to indicate that severe COVID-19 is associated with enhanced granule mobilization in NDN, except for the granule subset requiring the most potent stimulus, azurophilic granules. LDN demonstrate an impaired *in vivo* response to degranulation, except for the granule subset most easily induced to undergo exocytosis, secretory vesicles.

We previously reported that an intermediate level of CD16 expression (CD16^Int^) identifies a distinct subpopulation of LDN that emerges in severe COVID-19 (16). CD16^Int^ LDN have an activated genetic and proteomic phenotype, and their prevalence in the circulation predicts disease severity. CD16^Int^ and CD16^Hi^ LDN obtained by cell sorting and NDN isolated by gradient centrifugation were available from 7 COVID-19 patients to compare expression of neutrophil granule markers. **Figure 2B** shows CD16^Hi^ LDN express CD35, CD11b, CD66b, and CD63 at the same levels as COVID NDN. On the other hand, CD16^Int^ LDN showed reduced CD35, CD11b, and CD63 expression, while CD66b expression was increased. We interpret these data to indicate that CD16^Int^ LDN represent a unique subpopulation of LDN with impaired mobilization of secretory vesicles, gelatinase granules and azurophilic granules, while specific granule mobilization is enhanced.

Previous studies found elevated levels of granule constituents in the plasma of COVID-19 patients (21, 31, 48–51). To confirm our granule marker results, constituents of gelatinase granules (MMP9), specific granules (lactoferrin), and azurophilic granules (human neutrophil elastase, HNE) were quantified in plasma from HD and severe COVID-19 patients. **Figure 2C** shows that all three markers were significantly increased in plasma from COVID-19 patients. Thus, plasma concentrations of granule contents and neutrophil plasma membrane expression of granule markers present consistent findings for gelatinase and specific granules, while CD63 expression failed to conform with azurophilic granule mobilization detected by HNE release into plasma.

### NET Formation

**Figure 3:**
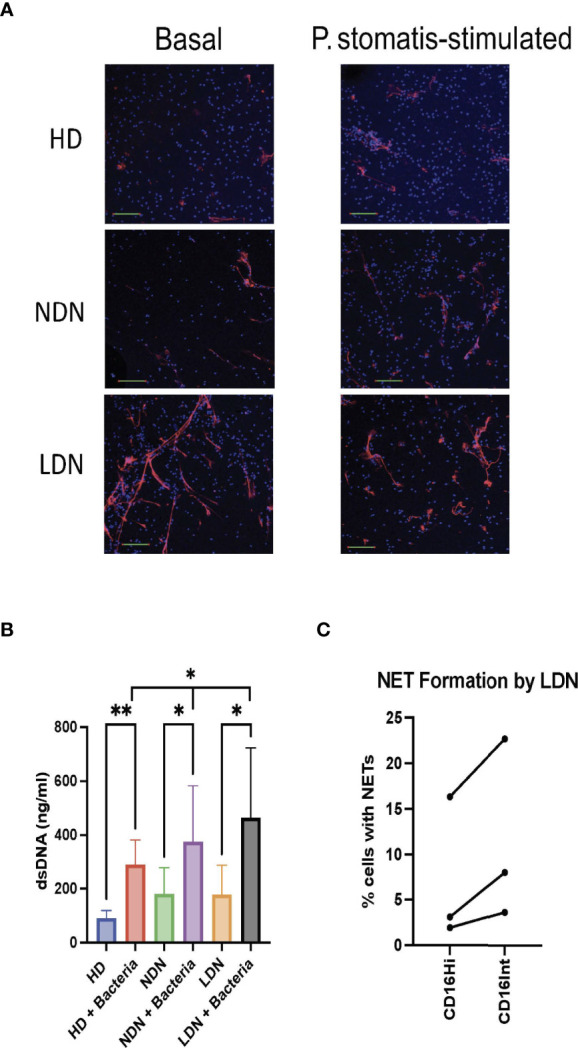
Enhanced NET formation by COVID-19 LDN. Panel **(A)** shows merged confocal images of lactoferrin staining of NETs (Red) and DAPI staining of DNA (Blue), demonstrating NET formation, in the absence (Basal) or presence of CFSE-labelled *P. stomatis*, in HD NDN, COVID NDN, and COVID LDN, representative of 4 experiments. Images shown are stacks of the entire collection of acquired z-planes at 20x magnification, scale bar=100 mm. Individual images are shown in **Supplemental Figures 1** and **2**. Panel **(B)** shows release of dsDNA into supernatant from basal or P. stomatis stimulated HD NDN, COVID NDN, and COVID LDN. NET formation by COVID LDN was significantly greater following bacterial stimulation, compared to COVID NDN and HD NDN, n=12-15. *p < 0.05, **p < 0.01. Panel **(C)** shows the results of 3 separate experiments comparing the percent of CD16^Hi^ LDN and CD16^Int^ LDN spontaneously forming NETs, visualized by confocal microscopy.

Enhanced NET formation by circulating neutrophils in COVID-19 is well established, and NETs are observed in the lungs of patients with COVID-19 ARDS and in areas of microthrombosis (10–17). We compared spontaneous and bacterially-stimulated NET formation by isolated HD NDN, COVID NDN and COVID LDN by confocal microscopy. **Figure 3A** shows a representative merged confocal microscopic image of lactoferrin and DAPI staining of NETs under basal and stimulated conditions, and individual images of each stain are shown in **Supplemental Figures 1, 2**. Minimal extracellular lactoferrin was observed directly adjacent to unstimulated HD NDN and COVID NDN. On the other hand, NETs were observed emanating from many unstimulated COVID LDN. Incubation with the emerging oral pathogen, *Peptoanaerobacter stomatis*, for 3 hr resulted in a greater number and larger patches of nets in COVID LDN, compared to either COVID NDN or HD NDN. Enlarged images confirming co-localization of extracellular staining for lactoferrin and DAPI, indicating lactoferrin and dsDNA co-localize outside of cells, are shown in **Supplemental Figures 3, 4**. To quantify NET release, extracellular dsDNA was measured in the same groups of cells. **Figure 3B** shows that although NET formation in unstimulated COVID LDN and COVID NDN was numerically greater than in HD LDN, those differences were not statistically significant. All three neutrophil groups showed a significant increase in NET formation following incubation with *P. stomatis* for 3 hr. The release of dsDNA was significantly greater following bacterial stimulation in COVID LDN, compared to either COVID NDN or HD NDN. Three sets of sorted CD16^Hi^ and CD16^Int^ LDN were available to compare NET formation in LDN subsets. As shown in **Figure 3C**, NET formation occurred in a greater number of CD16^Int^ LDN for each set, however, those differences were not statistically different in this limited data set. Taken together, our data suggest that COVID LDN possess a higher capacity for stimulated NET formation, and NET formation may be greater in CD16^Int^ LDN than CD16^Hi^ LDN.

### T-Cell Suppression

**Figure 4:**
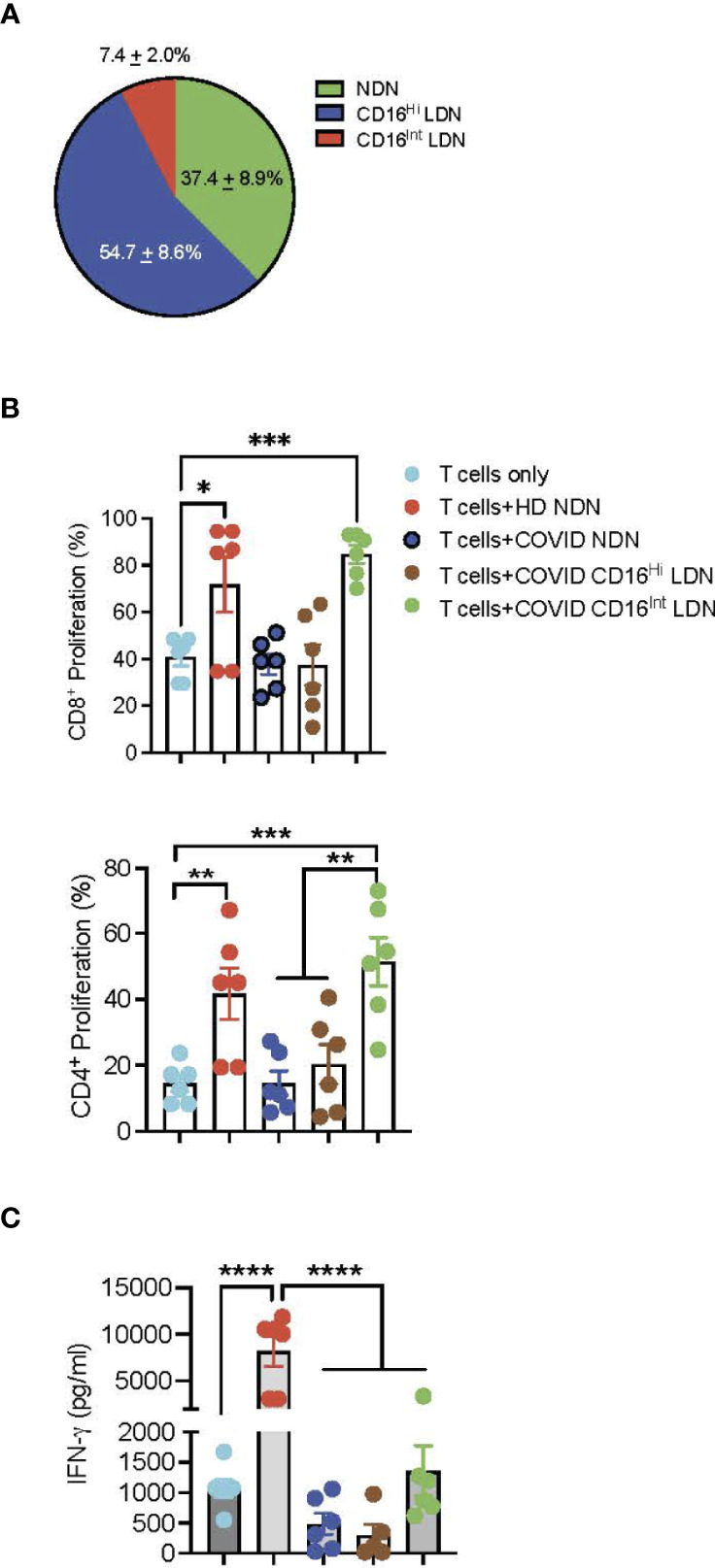
T cell activation assay. **(A)** Pie chart showing the distribution of NDN, CD16^Hi^ and CD16^Int^ LDN from severe COVID-19 patients (n=12). **(B)** T cells from the same donor were surface labeled with CellTrace CFSE dye and then co-cultured with ND NDN, COVID NDN, CD16^Hi^ LDN, or CD16^Int^ LDN for 4 days in the presence of plate-bound anti-CD3. Cells were collected and stained with CD4 or CD8. T cell proliferation was determined by flow cytometry and is expressed as mean ± SEM percent proliferation (n=6). **(C)** Supernatants from those 6 experiments were harvested and the IFN-γ level was determined by ELISA. Results are expressed as mean ± SEM in pg/ml. *p < 0.05, **p < 0.01,***p < 0.001, ****p < 0.0001.

The genetic signature of LDN has been interpreted to indicate at least a portion of this neutrophil population has immunosuppressive activity (23, 25). Evaluation of neutrophil supernatants or neutrophil-T cell interaction showed impairment of T cell proliferation (52, 53). To determine the effect of different neutrophil populations on T cell activation, we determined the effect of NDN and CD16^Hi/Int^ LDN from 6 COVID-19 patients or HD NDN on T cell activation. T cells from a single donor were labeled with CellTracker dye CFSE and then incubated with NDN, CD16^Hi^ LDN, or CD16^Int^ LDN from COVID-19 patients or HD NDN. The distribution of those 3 neutrophil populations in 12 COVID-19 patients is shown in **Figure 4A**. NDN accounted for 37.4 ± 8.9% of total neutrophils, CD16^Hi^ LDN for 54.7 ± 8.6, and CD16^Int^ LDN for 7.4 ± 2.0%. T cell proliferation was assessed based on CFSE dilution, and cell culture supernatants were harvested to measure IFN-γ levels. While co-culture of CD16^Int^ LDN from COVID-19 patients stimulated proliferation of CD4^+^ and CD8^+^ T cells to the same degree as HD NDN, CD16^Hi^ LDN and COVID NDN failed to stimulate CD8^+^ and CD4^+^ T cell proliferation (**Figure 4B**). Co-culture with HD NDN significantly stimulated IFN-γ production. On the other hand, COVID NDN, CD16^Int^ LDN, and CD16^Hi^ LDN all failed to stimulate IFN-γ production (**Figure 4C**).

## Discussion

Our study provides additional context to the expanding literature implicating neutrophils in the pathogenesis of COVID-19 ARDS. Gene and protein signatures define multiple clusters of mature and immature neutrophils emerging in the PBMC fraction (LDN) from severe COVID-19 patients (16, 22, 23, 25, 27, 28). Those LDN are present in BALF, as well as in the circulation (16, 22). An altered functional status of those neutrophil clusters is inferred from their gene and protein signatures, leading to the hypothesis that dysregulated neutrophil host defense mechanisms and adaptive immune system suppression are major contributors to progression to severe COVID-19 (25, 26, 45, 54). To characterize the dysregulated neutrophil functional responses associated with COVID-19 ARDS, the present study evaluated host defense functions and suppression of T cell activation of NDN and two populations of LDN from COVID-19 patients. The major finding of our study is that neutrophil populations from patients with severe/critical COVID-19 are functionally distinct from each other and from NDN of healthy donors. COVID NDN exist in a primed state, but they failed to support T-cell activation. LDN are generally hyporesponsive, except for more robust NET formation. Separating LDN by their CD16 expression defines subsets with distinct activation and immunosuppressive signatures.

NDN account for an average of 37% (range 1% to 83%) of total neutrophils in our severe/critical COVID-19 patients. They are primed for enhanced phagocytosis, respiratory burst activity, and degranulation. Consistent with our findings, Masso-Silva et al. (8) reported increased basal and stimulated ROS production and increased phagocytosis by neutrophils isolated from COVID-19 patients. On the other hand, Schulte-Schrepping et al. (25) reported that neutrophils from severe COVID-19 patients showed reduced respiratory burst activity, while phagocytosis did not differ from healthy donors. Our findings with granule plasma membrane markers and plasma concentrations of granule cargo suggest robust degranulation of secretory vesicles, gelatinase granules, and specific granules. The presence of elevated levels of HNE in patient plasma, while expression of the plasma membrane marker of azurophilic granules, CD63, did not increase, may be due to the hierarchical nature of neutrophil granule exocytosis, in which fewer than 10% of azurophilic granules are mobilized during an inflammatory response (55, 56). Previous studies of neutrophil degranulation in COVID-19 described conflicting results, depending on the method of analysis and experimental conditions. Using granule marker expression, exocytosis of specific and gelatinase granules was increased, unchanged, or decreased in severe COVID-19, while azurophilic granules were uniformly mobilized (9, 28, 29, 31, 32, 36, 46). On the other hand, reports of elevated concentrations of granule cargo in blood and BALF suggest that azurophilic, specific, and gelatinase granules all undergo exocytosis *in vivo* in COVID-19 (21, 48–51). Taken together, we suggest that NDN from COVID-19 patients undergo *in vivo* exocytosis of all granule subsets in the hierarchical manner previously described (55, 56), leading to release of toxic granule cargo and priming of respiratory burst activity (57, 58).

LDN are present in a number of disease states, including cancer, sepsis, and autoimmune disorders (59–62), and small numbers of LDN were recently reported in healthy individuals (63). LDN account for an average of 17% of PBMCs in systemic lupus erythematosus (SLE) patients (range 1.2% to 54%) (62), and the number of LDN correlates with disease activity (64). Significant heterogeneity of LDN functional responses appears to be disease dependent (59, 60), and LDN may represent primed, mature neutrophils in healthy individuals (63). Mistry et al. (65) described reduced NET formation, chemotaxis, and phagocytosis in CD10^-^ LDN, compared to CD10^+^ LDN in SLE, while degranulation was enhanced in both LDN populations. LDN from SLE patients are not immunosuppressive (66), while both immunosuppressive and antitumor LDN phenotypes are described in cancer (59). LDN are reported to account for 20% to 50% of neutrophils in COVID-19 ARDS and 5% to 20% in moderate COVID-19 and non-COVID ARDS (16, 22, 25, 28, 31, 53). Consistent with those reports, LDN account for over 50% of neutrophils in our severe/critical COVID-19 patients, and functional activities differed between LDN and NDN. COVID LDN were not primed for increased phagocytosis, respiratory burst activity, or degranulation. Secretory vesicles showed increased mobilization, which may be explained by their sensitivity to stimuli leading to the earliest and most complete mobilization (55, 56). The responses to *in vitro* incubation with TNFa indicate an impaired capability of LDN to undergo priming, as only respiratory burst activity and exocytosis of specific granules are enhanced. Previous studies reported increased neutrophil expression of genes encoding proteins involved in neutrophil degranulation in COVID-19, interpreted as indicating enhanced granule mobilization (23–25, 29). Proteins destined to be incorporated into granules are produced during different phases of neutrophil maturation (67). Our results suggest that gene expression of granule constituents in COVID LDN may be due to the presence of immature neutrophil progenitors with incomplete granule formation and does not necessarily predict granule mobilization. A number of studies report increased LDN plasma membrane expression of CD63 and CD66b, compared to NDN (9, 28, 29), while CD11b expression in COVID LDN varied (27, 28, 31, 32, 36, 46). The ‘targeting by timing hypothesis’ predicts granule heterogeneity results from protein synthesis at the time individual granule subsets are formed (67). Thus, granule marker expression is impacted by the proportion of mature and immature neutrophils in various LDN clusters.

Evidence supports NET formation as critical in the development of ARDS and immunothrombosis in COVID-19 patients (10–17). The present study found spontaneous and stimulated NET formation was enhanced in COVID LDN, compared to COVID NDN and HD NDN. NET formation by COVID NDN was numerically, but not statistically, higher than that of HD NDN. Our results are consistent with previous studies showing COVID-19 neutrophils exhibit enhanced spontaneous and stimulated NET formation (12, 13, 15, 16). Our study suggests that LDN are primarily responsible for NET formation in COVID-19, similar to LDN in SLE (61, 65).

We recently reported that expression of intermediate levels of CD16 identifies a unique population of LDN that emerges in severe COVID-19, and that the proportion of CD16^Int^ LDN predicts clinical outcome (16). On average CD16^Int^ LDN accounted for 13% (range 1% to 34%) of LDN in our patients. While sorted cells were not available for phagocytosis and respiratory burst assays in the present study, we reported previously that CD16^Int^ LDN show enhanced phagocytosis, as well as enhanced platelet activation and cytokine production (16). In the present study CD16^Int^ LDN showed reduced secretory vesicle, gelatinase granule, and azurophilic granule exocytosis, while, surprisingly, specific granule exocytosis was enhanced. Although our analysis was limited by the availability of only 3 samples, CD16^Int^ LDN appear more likely to form NETs upon stimulation than CD16^Hi^ LDN. This observation is consistent with our previous study showing spontaneous *in vitro* NET formation by CD16^Int^ LDN (16). Thus, our results support CD16^Int^ LDN as a unique subset of neutrophils in COVID-19 expressing of both enhanced and impaired pro-inflammatory functional responses.

In a number of diseases LDN contain granulocytic myeloid-derived suppressor cells (G-MDSC) that suppress the adaptive immune response by inhibiting proliferation of T-cells, promoting T-cell anergy, and recruiting regulatory T (Treg) cells (68, 69). Single cell RNA sequencing analysis identified a cluster of circulating LDN with characteristics of G-MDSC that are increased in severe COVID-19 (25–27, 53, 54). Cultivation of activated T cells with COVID-19 LDN or their supernatants inhibit T cell proliferation and impair IFN-γ production (27, 38, 53, 54). The current study found that CD16^Hi^ LDN and COVID NDN failed to support T-cell proliferation, compared to HD NDN. On the other hand, CD16^Int^ LDN promoted T cell proliferation to a level similar to HD NDN. These findings suggest that the proportion of different neutrophil subsets in the lung or circulation determine the level of T-cell proliferation and activation. Thus, changing proportions of neutrophil subsets over the course of COVID-19 may play a role in disease outcome. All 3 neutrophil populations from critically ill COVID-19 patients suppressed stimulation of IFN-γ production, compared to HD NDN. A relationship between neutrophil degranulation and T-cell activation has been established (70–73), providing one potential explanation for the differential T-cell regulation by neutrophil populations in COVID-19. We interpret these results to show differential regulation of the adaptive immune response by different neutrophil populations in COVID-19 patients, while IFN-γ release from effector T-cells is inhibited by all neutrophil populations.

We acknowledge several weaknesses in our study. We could not determine the influence, if any, of corticosteroid therapy on functional changes of neutrophil subsets in COVID-19, as most patients in our cohort received corticosteroids. Recent studies reported disparate results on the effect of steroid treatment on neutrophil functions (28, 74). Many of our patients were recruited when the delta variant was the predominant cause of infection, however, the SARS-CoV-2 variant infecting the patients in our study was not determined. Thus, we cannot conclude that all SARS-CoV-2 variants induce similar changes in neutrophil population functional responses. The time at which blood was obtained after infection with SARS-CoV-2 was not uniform, averaging 8.5 days after hospitalization, and longitudinal sampling was not performed. Due to limitations in the amount of blood obtained from each patient, each functional analysis was only performed on a fraction of patients from the entire cohort. Additionally, the percent of CD16^Int^ LDN varies over time (16), and the distribution of NDN, CD16^Int^ LDN, and CD16^Hi^ LDN in the present study is based on results from a fraction of patients from our cohort. Thus, we could not determine possible relationships between functional responses and neutrophil subset distribution, comorbidities, and patient outcome, nor determine the timing of neutrophil functional changes relative to the onset of ARDS. None of the neutrophil granule plasma membrane markers is restricted to a single granule subset. For example, analysis of granule constituents by Rorvig et al. (75) shows that the distribution of CD63 is 79% in azurophilic granules, 14% in specific granules, and 5% in gelatinase granules, while CD11b distribution is 2% in azurophilic, 37% in specific, 43% in gelatinase granules.

In summary, our study shows that functional capabilities of NDN and LDN differ significantly in COVID-19 patients. NDN are primed for enhanced phagocytosis, respiratory burst, and degranulation, while NET formation and stimulation of T-cell activation are impaired. The presence of both activated and immunosuppressive functions by COVID NDN are consistent with the gene signatures described by Auschenbrenner et al. (23). In contrast, priming and activation of COVID LDN are impaired, with the notable exception of NET formation. We previously reported emergence of a unique subset of LDN, CD16^Int^ LDN, that expresses a pro-inflammatory gene signature (16). CD16^Int^ LDN are functionally unique in that they show enhanced phagocytosis, NET formation, platelet activation, cytokine production, and T-cell activation, while degranulation is impaired. Our data suggest the hypothesis that different neutrophil populations possess complementary pro-inflammatory and immunosuppressive functional signatures, and those neutrophil populations may cooperate to produce ARDS and hypercoagulation in severe COVID-19.

## Materials and Methods

### Patient Recruitment

The Institutional Review Board at University of Louisville approved the present study and written informed consent was obtained from subjects or their legal authorized representatives (IRB No. 20. 0321). Patients were recruited between November, 2020 and December, 2021. Inclusion criteria were all hospitalized adults (older than 18) who have positive SARS-CoV-2 test results and were consented to this study. Exclusion criteria included age younger than 18 and refusal to participate. COVID-19 patients enrolled in this study were diagnosed with a 2019-CoV detection kit using real-time reverse transcriptase–polymerase chain reaction performed at the University of Louisville Hospital Laboratory from nasal pharyngeal swab samples obtained from patients. The identification of COVID-19 patients with severe/critical disease was based on the requirement for high-flow oxygen support or the need for mechanical ventilation and this group had blood draw along with their standard laboratory work. All COVID-19 patients were followed by the research team daily and the clinical team was blinded to findings of the research analysis to avoid potential bias.

The demographic characteristics (age, sex, height, weight, Body Mass Index (BMI) and clinical data (symptoms, comorbidities, laboratory findings, treatments, complications, and outcomes) were collected prospectively. All data were independently reviewed and entered into a computer database. For hospital laboratory complete blood count (CBC) tests, normal values are the following: white blood cell (4.1-10.8 x10^3^/μL); hemoglobin (13.7-17.5g/dL); platelet (140-370 x10^3^/μL). For hospital laboratory inflammatory and coagulation markers, normal values are the following: D-dimer (0.19-0.74 μg/ml Fibrinogen Equivalent Units); ferritin (7-350 ng/ml); lactate dehydrogenase (LDH) (100-242 Units/Liter); C-reactive protein (<3.0 mg/L).

### Neutrophil Isolation

**Table 1** shows that blood for neutrophil isolation was collected an average of 8.5 days (median 6.5 days) after hospitalization (range 1 to 36 days). Blood was collected into citrate. Neutrophils were isolated from the blood of healthy donors using plasma-Percoll gradients as previously described (76, 77). Microscopic evaluation of isolated cells showed that >92% of cells were neutrophils. Trypan blue exclusion indicated that >95% of cells were viable. Neutrophils isolated by gradient centrifugation were used in flow cytometric experiments examining phagocytosis, H_2_O_2_ generation, and exocytosis, as neutrophils were specifically gated. The Institutional Review Board of the University of Louisville approved the use of human donors who provided informed consent (IRB No. 96.0191).

Alternatively, neutrophils were isolated from whole blood of healthy donors and COVID-19 patients using an EasySep Direct Human Neutrophil Isolation Kit (STEMCELL Technologies, Cat# 19666) accordingly to the manufacturer’s instructions and >95% purity was obtained. The purified PMNs were resuspended in colorless RPMI and experiments were performed at 37°C in 5% CO_2_. Neutrophils isolated by this method were used in NET assays.

### Isolation of CD16 LDN by Cell Sorting

PBMCs were isolated from peripheral blood using a Ficoll gradient centrifugation (Lympholyte^®^-H solution, CEDARLANE) followed by isolation of the buffy-coat layer. The cells were then washed using complete RPMI-1640 (cRPMI) (Sigma). The cell pellet was washed with sterile, cold PBS and added with Fc block (Human TruStain FcX™, Biolegend) to incubate at 4°C for 10 minutes followed by staining with anti-human monoclonal antibodies (mAb) for CD66b-PE (Clone G10F5, Biolegend) and CD16-APC (Clone 3G8, Biolegend) and with the viability dye eFluor 780 (eBioscience), then incubated at 4°C for 20 minutes in dark. The cells were washed with 3-5 ml of MACs buffer (Miltenyi Biotech) by centrifugation at 500 g for 5 minutes. The pellet was then suspended in MACs buffer, filtered, and the cells sorted with a BD FACsAria III sorter to collect the CD16^hi^ (gated as viable, CD66b^+^CD16^hi^) and CD16^int^ LDNs (gated as viable, CD66b^+^CD16^int^) using separate collection tubes containing a mixture of 50% Fetal Bovine Serum (Atlanta Biologicals), 40% PBS (Sigma) and 10% HEPES buffer (Corning). After collection, the collection tubes were centrifuged at 500 g at 4°C for 8 minutes, washed once with cRPMI, counted and resuspended in required volumes of cRPMI.

### Phagocytosis and H_2_O_2_ Generation

To measure H_2_O_2_ production, isolated neutrophils or PBMCs (4 x 10^6^ cells/ml) were incubated with 2’,7’-dichlorofluorescin diacetate (final concentration 0.5 mM; Molecular Probes/Invitrogen, Carlsbad, CA, USA) for 10 min at 37°C. Fifty microliters of cell suspension was sampled before, and 10 min after, the addition of 50 ml of opsonized, propidium iodide-labeled *Staphylococcus aureus* (final concentration ~10^8^ bacteria/ml). Samples were analyzed for uptake of labeled bacteria and oxidation of 2’,7’-dichlorofluorescin diacetate to fluorescent 2’,7’-dichlorofluorescein by flow cytometry as previously described (57, 76). The LDN population in the PBMC fraction was defined for analysis by gating on CD66b^+^ cells.

### Degranulation

Exocytosis of secretory vesicles, gelatinase granules, specific granules, and azurophilic granules in HD NDN, COVID NDN, and COVID LDN was determined by measuring the increase in plasma membrane binding of FITC-conjugated monoclonal anti-CD35 (clone E11; Pharmingen, San Diego, CA, USA), ALEXA-Fluor 488-conjugated monoclonal anti-CD11b (BD Biosciences, San Jose, CA, USA), FITC-conjugated monoclonal anti-CD66b (clone CLB-B13.9; Accurate Chemical, Westbury, NY, USA), and FITC-conjugated anti-CD63 (clone AHN16.1/46-4-5; Ancell Corporation, Bayport, MN, USA), respectively, on 4 x 10^6^/ml neutrophils using a FACSCalibur flow cytometer (Becton Dickenson, Franklin Lakes, NJ, USA) as previously described (47, 57). The LDN population in the PBMC fraction was defined for analysis by gating on CD66b^+^ cells.

For low density neutrophil subsets, Single cell suspensions of PBMCs/Neutrophils were washed with 1 ml PBS (SIGMA) by centrifugation at 500 g for 5 minutes at 4°C. The pellets were then stained with anti-human CD66b-PE (Clone G10F5, Biolegend), anti-human CD16-APC (Clone 3G8, Biolegend), anti-human CD35-FITC (Clone 9H3, Biolegend), anti-human CD11b-PE-Cy7 (Clone CBRM1/5, Biolegend), anti-human CD63-APC-Cy7 (Clone H5C6, Biolegend) and anti-human CD10-PerCP-Cy5.5 (Clone HI10a, Biolegend) monoclonal antibodies, briefly vortexed and incubated for 30 minutes at 4°C in dark. After incubation, cells were washed with ice-cold PBS by centrifugation at 500 g for 5 minutes at 4°C. The pellets were finally suspended in 250-300 µl of cold PBS, briefly vortexed and acquired by BD FACsCanto for analysis.

Plasma concentrations of constituents of gelatinase granules (MMP9, R&D Systems, Minneapolis, MN, USA), specific granules (lactoferrin, Abcam, Cambridge, MA, USA), and azurophilic granules (HNE, Abcam, Cambridge, MA, USA) were determined by commercial ELISA kits, according to the manufacturers’ instructions.

### Bacterial Preparation

*Peptoanaerobacter stomatis* strain CM2 (provided by Dr. Slava Epstein, Northeastern University) was cultivated in tryptic soy broth (TSB) supplemented with 20 g/L yeast extract, 1.0% hemin and 1.0% reducing agent (37.5 g/L NH_4_Cl, 25 g/L MgCl_2_ x 6H_2_O, 5 g/L CaCl_2_ x 2H_2_O, 50 g/L L-cysteine HCl, 5 g/L FeCl_2_ x 4H_2_O) overnight at 37°C under anaerobic conditions as previously described (78). Overnight culture of *P. stomatis* was diluted in fresh growth media and incubated under anaerobic conditions for 4-5 hours until reaching an OD_600_ between 0.4 to 0.6. For confocal assays, *P. stomatis* was labeled with carboxyfluorescein succinimidyl ester (CFSE, 0.25mg/ml; Invitrogen, USA) for 30 min at room temperature in the dark and washed 3 times with PBS prior to its use.

### NET Formation

For confocal imaging, neutrophils 1 × 10^6^ cells/experimental condition in RPMI media without phenol red supplemented with 10mM HEPES were seeded onto poly-L-Lysine-coated 12 mm glass coverslips that were placed in 24-well dishes. Cells were allowed to attach for 30 minutes at 37°C, after which, CFSE-labeled *P. stomatis* (10 MOI) was added to appropriate wells and the plate centrifuged for 10 min at 600 rcf to synchronize phagocytosis. Untreated and treated cells were incubated for an additional 3h at 37°C. After fixation with paraformaldehyde (2% final concentration) for 30 min at room temperature, cells were blocked in 1% BSA/PBS for 1h at room temperature, followed by incubation with anti-lactoferrin antibody (MP Biomedicals #55040; 1:500), in 1% BSA/PBS, overnight at 4°C. Primary antibody was washed off with three 5 min PBS washes, followed by incubation with donkey anti-rabbit Alexa-Fluor 546 (Thermo Fisher #A10040; 1:1000) in 1% BSA/PBS for 1h at room temperature. Nuclei were stained with DAPI (4′,6-diamidino-2-phenylindole), and coverslips were mounted onto glass slides using ProLong Gold Antifade reagent (Thermo Fisher). Images were acquired using an Olympus Fluoview FV-1000 confocal coupled to an Olympus 1X81 inverted microscope, 20× objective, and FV-10 ASW 2.1 software. A multi-channel scanning configuration with sequential line scanning was setup for acquisition of DAPI, FITC (*P.stomatis* staining), and AF546 (lactoferrin immunostaining). Optimal brightness setting for each channel was configured to yield maximal intensity without saturation and exclusion of non-specific emission. Images from all planes of cells were acquired and assembled z-stacks of the planes are presented.

For double-stranded DNA (dsDNA) quantification, neutrophils (4×10^6^) were resuspended in 1 ml of colorless RPMI-1640 media. The neutrophils were unstimulated or stimulated with *P. stomatis* at multiplicity of infection (MOI) of 50. Aliquots of 250 µl of cells (1×10^6^) were plated in triplicate in 96 well plate followed by centrifugation at 600xg at 14°C for 4 mins. The plate was then incubated for 3 h at 37°C, 5% CO_2_. After incubation the plate was centrifuged at 800 g at 4°C for 2 min and supernatants were transferred to tubes and stored at 4°C until use. The supernatants were quantified using Quant-iT PicoGreen dsDNA assay kit (Invitrogen, USA). The samples were incubated with equal volume of 1X PicoGreen 5 min at room temperature and protected from light. The plates were excited at 485 nm, and the fluorescence emission intensity was measured at 520 nm using a spectrofluorometer (SpectraMax M2; Molecular Devices).

### T Cell Suppression of Proliferation and IFN-γ Release Assay

T cells were isolated from healthy human PBMCs, Fc blocked, and surface stained with viability dye eFluor 780 (eBioscience) and anti-human CD3-PerCp-Cy5.5mAb (Clone HIT3a, Biolegend) as mentioned earlier. The cells were then washed with 5 ml of sterile MACs running buffer (Miltenyi Biotech), filtered, and resuspended in appropriate volume of MACs buffer for acquisition by BD FACsAria III sorter. The T cells were gated as viable and CD3+ cells. The isolated T cells were washed (500 g for 5 minutes using a room temperature centrifuge) with pre-warmed 0.1% Bovine Serum Albumin (BSA, Equitech-Bio, Inc). The pellet was then suspended in appropriate volume of pre-warmed 0.1% BSA (1-10 x 10^6^ cells/ml) and CFSE (1µM/ml) (Cell Trace™ CFSE Cell Proliferation Kit, eBioscience) was added to the cell suspension, gently mixed and incubated at 37°C for 10 minutes with gentle mixing 2-3 times during the incubation. Upon the completion of incubation, the CFSE labeling was stopped by addition of 5 ml of ice-cold cRPMI and incubation for 5 minutes on ice and centrifugation at 500 g for 5 minutes at 4°C. The CFSE-labelled T cells were then suspended in appropriate volume of cRPMI and added to U-bottomed 96 well plates (100 µl) pre-coated with anti-human CD3 antibody (Clone OKT3,1µg/ml in PBS at 4°C overnight followed by gentle washing with sterile PBS) to which 100 µl of neutrophils (a ratio of 1:1) was added, gently mixed and incubated at 37°C with 5% CO_2_ for 4 days. After 4 days, the culture supernatants were collected and stored at -20°C until analyzed. The cells were harvested, washed, Fc-blocked and stained for surface markers. The cells were stained for viability dye eFluor 780 (eBioscience), anti-human CD4-PE-Dazzle™ 594 (Clone A161A1, Biolegend) and anti-human CD8-APC (Clone SK1, Biolegend)mAb and acquired using FACsCanto for analysis of the T cell suppression. The supernatants were used to perform ELISA using a human IFN-γ ELISA Kit (Biolegend). ELISA was performed as per the instructions provided in the Kit protocol.

### Statistical Analysis

Results are shown as mean and standard error of the mean (SEM), unless otherwise stated. Analysis of groups was by analysis of variance using SPSS V27 (IBM, Chicago). The dependent variables were phagocytosis, H_2_O_2_, CD35, CD11b, CD66b, or CD63 and the independent factors were basal and stimulated. In a separate analysis we performed a repeated measures analysis on basal and *P. stomatis* stimulated NET formation for the groups HD NDN, COVID LDN, and COVID NDN. A *post hoc* analysis was performed using a one way analysis of variance comparing NET formation between groups. Difference was determined at p=0.05 using Student-Newman-Keuls. Comparison of 2 data sets was performed by Student t-test using GraphPad Prism v7.0 (La Jolla, California) with significance defined as *p* < 0.05.

## Data Availability Statement

The raw data supporting the conclusions of this article will be made available by the authors, without undue reservation.

## Ethics Statement

The Institutional Review Board at University of Louisville approved the present study and written informed consent was obtained from subjects or their legal authorized representatives (IRB No. 20. 0321 and IRB No. 96.0191). The patients/participants provided their written informed consent to participate in this study.

## Author Contributions

Study design and supervision: KM, JY, JH, SU, and MR. Sample collection, data acquisition, experimental work: RS, AV, MB, ML, XH, OC, CW, TS, SB, JI, CH, ST and TM. Data analysis, interpretation, and visualization: MB, KM, JY, SU, MR, and MB. Manuscript writing, review and editing: KM, RS, AV, MR, MB, SU, JH, and JY. All authors contributed to the article and approved the submitted version.

## Funding

This work was supported in part by a grant from the Jewish Heritage Fund for Excellence Research Enhancement Grant Program and the Endowment in Translational Research at the University of Louisville School of Medicine (JY). JH is supported by National Center For Advancing Translational Sciences [U18TR003787]; the National Institute of Environmental Health Sciences [P30 (P30ES030283)]; and Gilead Sciences COMMIT COVID-19 RFP Program [IN-US-983-6063].

## Author Disclaimer

The content is solely the responsibility of the authors and does not necessarily represent the official views of the National Institutes of Health or Gilead Sciences.

## Conflict of Interest

The authors declare that the research was conducted in the absence of any commercial or financial relationships that could be construed as a potential conflict of interest.

## Publisher’s Note

All claims expressed in this article are solely those of the authors and do not necessarily represent those of their affiliated organizations, or those of the publisher, the editors and the reviewers. Any product that may be evaluated in this article, or claim that may be made by its manufacturer, is not guaranteed or endorsed by the publisher.
