# Supplementary material for: Differential Functional Responses of Neutrophil Subsets in Severe COVID-19 Patients
Source: Front Immunol. 2022 May 31;13:879686. doi: 10.3389/fimmu.2022.879686 (PMC9197482; doi:10.3389/fimmu.2022.879686)
Supplement: Supplementary file 1 [file DataSheet_1.pdf]

Supplemental Figure 1

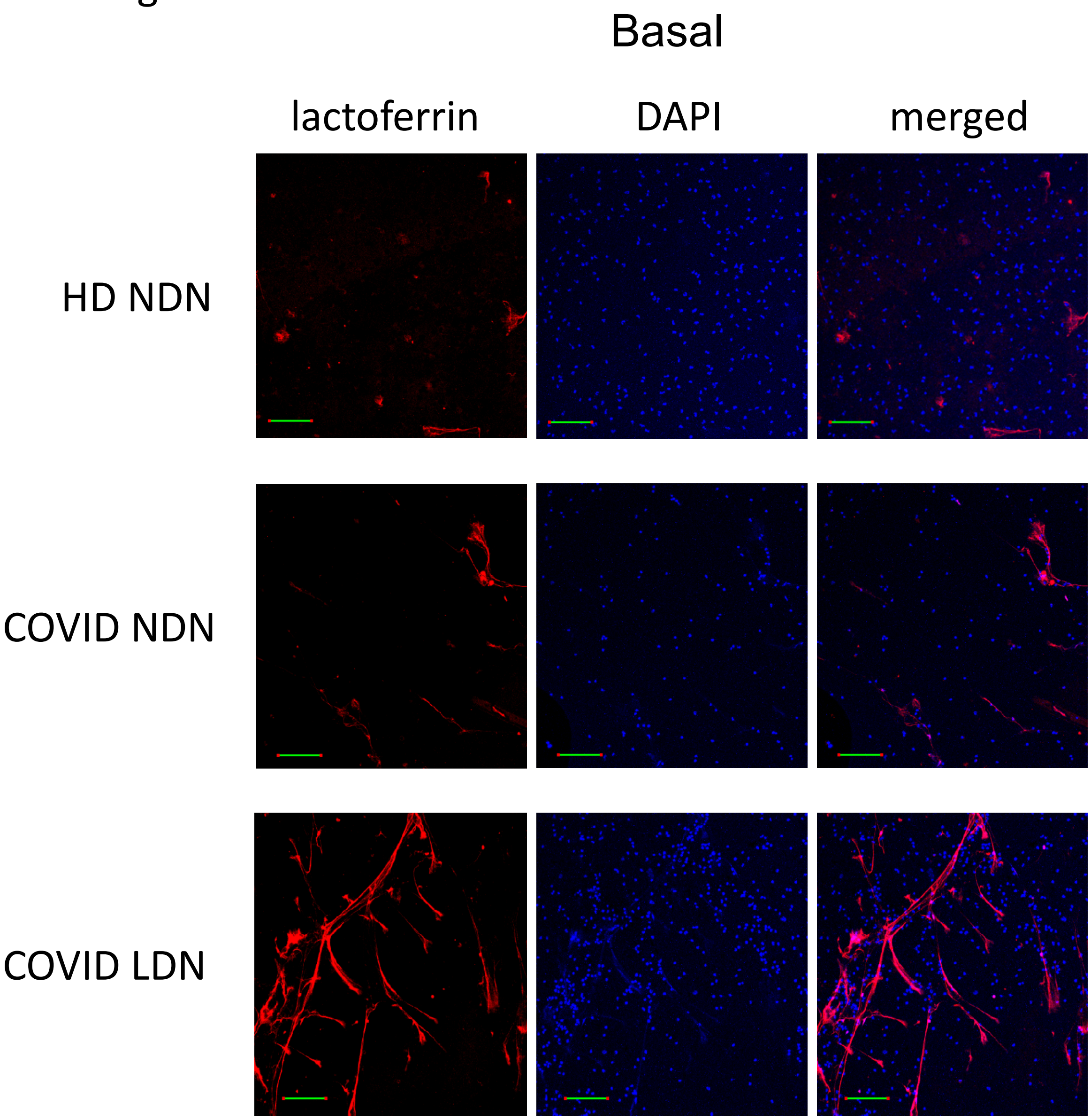

Supplemental Figure 1. Enhanced Basal NET formation by COVID LDN. Confocal images of staining for lactoferrin (Red) and DAPI (Blue), and merged images, of unstimulated (Basal) HD NDN, COVID NDN, and COVID LDN. Increased extracellular staining indicative of enhanced NET formation is present in COVID LDN images. Images shown are stacks of the acquired z-planes at 20x magnification, scale bar=100 μm.

Supplemental Figure 2

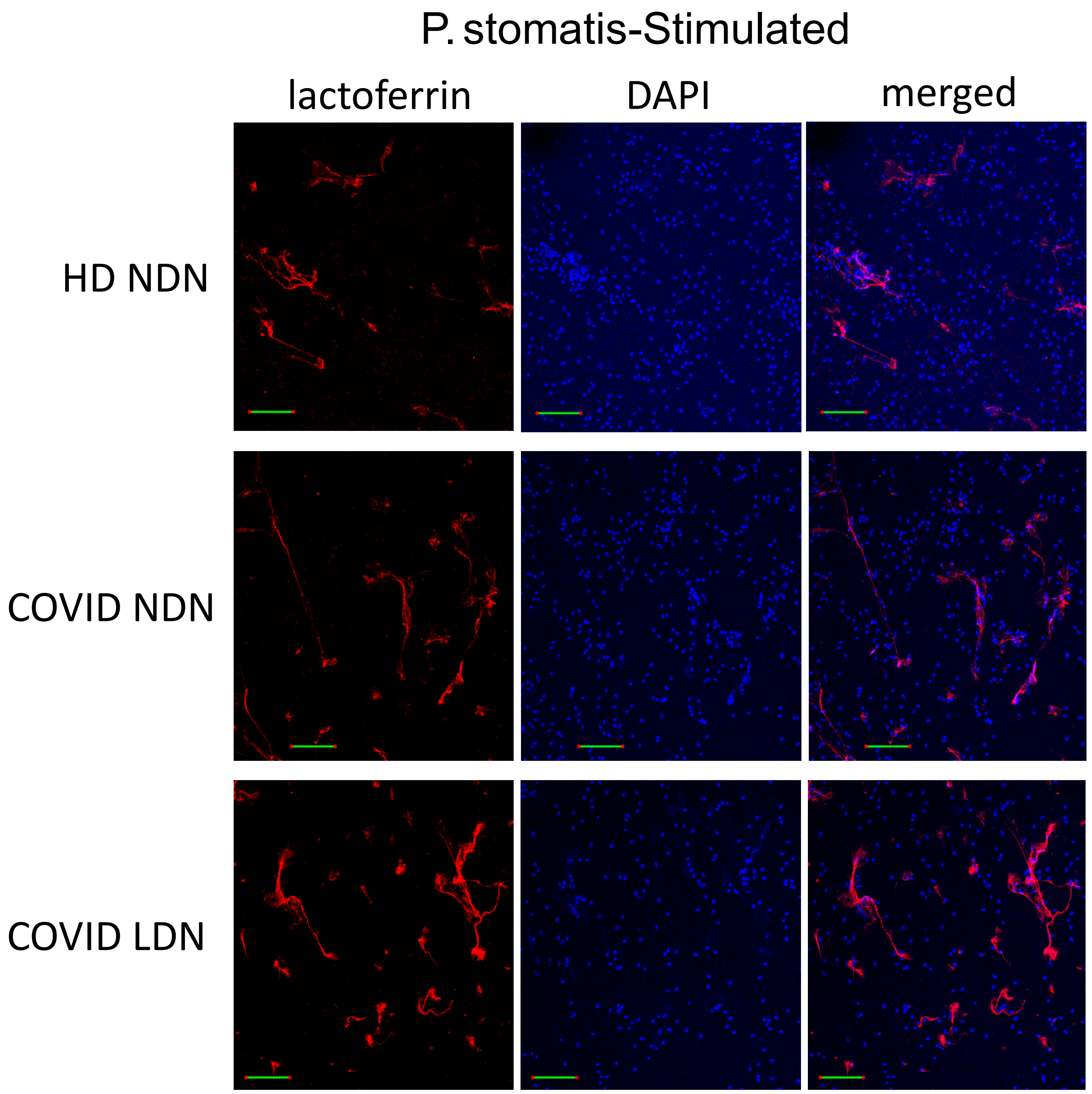

Supplemental Figure 2. Enhanced P. *stomatis*-stimulated NET formation by COVID LDN. Confocal images of staining for lactoferrin (Red) and DAPI (Blue), and merged images, of HD NDN, COVID NDN, and COVID LDN incubated with P. *stomatis* for 3 h. Increased extracellular staining indicative of enhanced NET formation is present in all neutrophil groups, compared to basal conditions. The number of NETs is increased in COVID LDN, compared to COVID NDN and HD NDN. Images shown are stacks of the acquired z-planes at 20x magnification, scale bar=100 μm.

Supplemental Figure 3

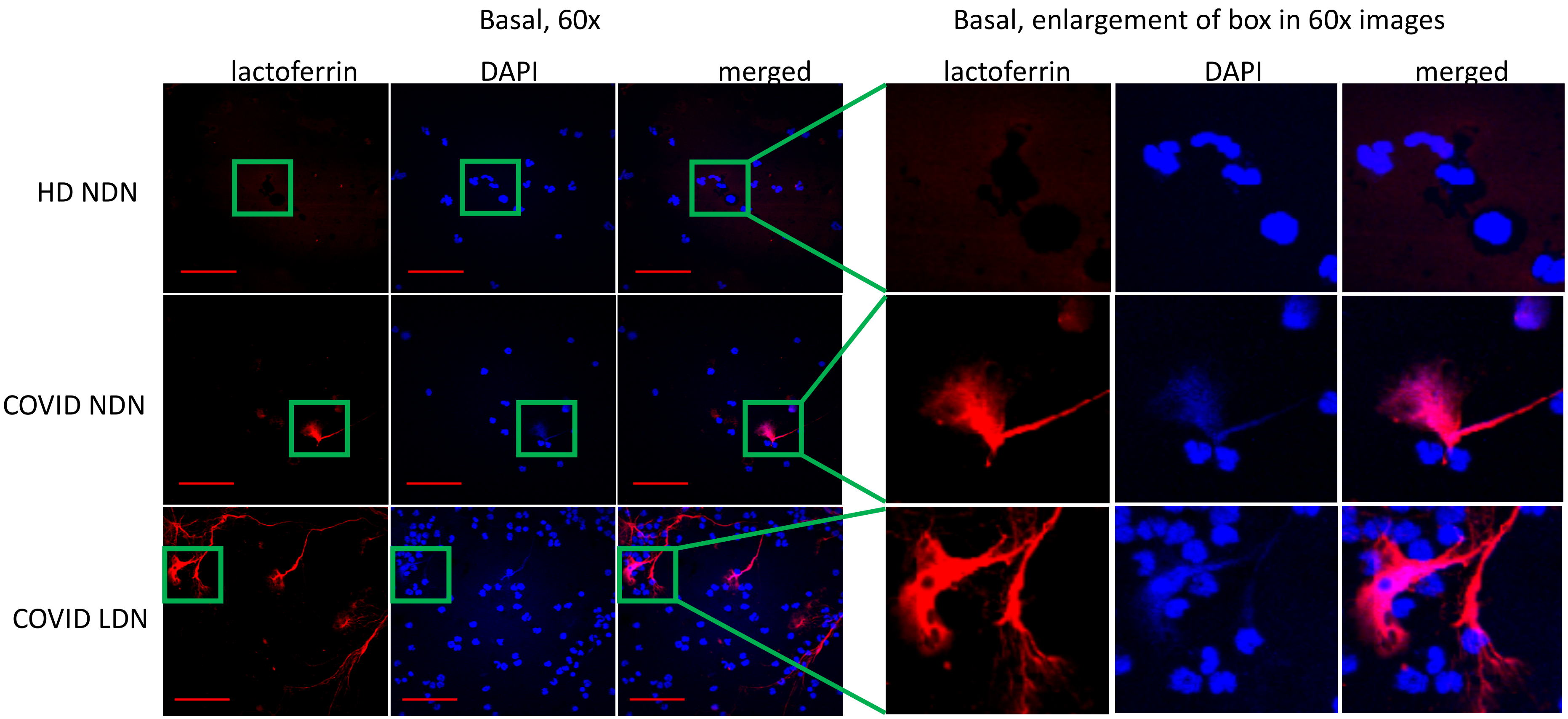

Supplemental Figure 3. Enlarged Confocal Images Confirming Basal NET formation. Confocal images of staining for lactoferrin (Red) and DAPI (Blue), and merged images, of unstimulated (Basal) HD NDN, COVID NDN, and COVID LDN. Images shown are stacks of the acquired z-planes at 60x magnification, scale bar=50 μm, and enlargements of the boxed areas. Extracellular lactoferrin staining co-localizes with DAPI staining of DNA, indicating the staining represents NETs.

Supplemental Figure 4

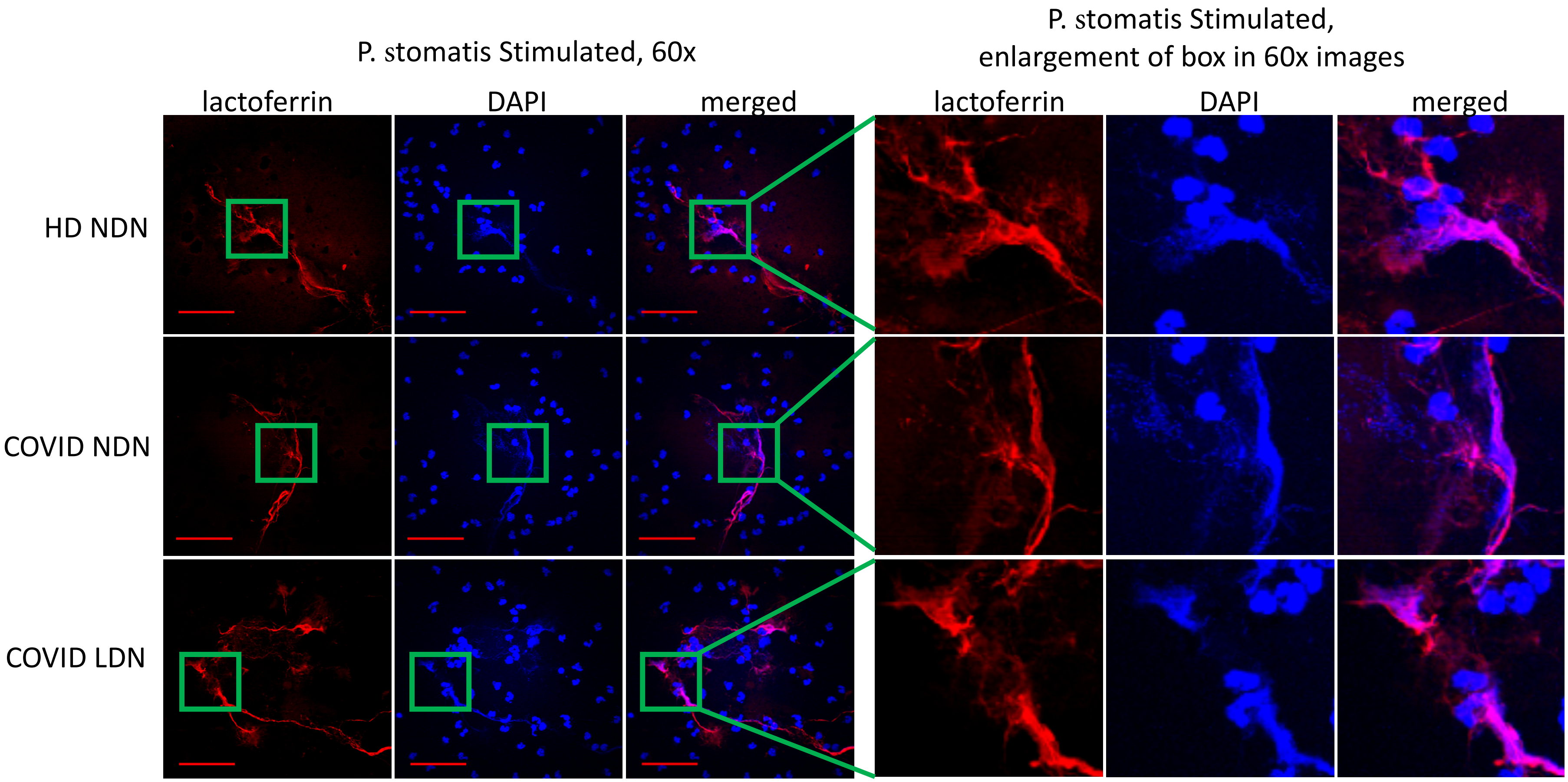

Supplemental Figure 4. Enlarged Confocal Images Confirming P. *stomatis*-stimulated NET formation. Confocal images of staining for lactoferrin (Red) and DAPI (Blue), and merged images, of HD NDN, COVID NDN, and COVID LDN incubated with P. *stomatis* for 3 h. Images shown are stacks of the acquired z-planes at 60x magnification, scale bar=50 μm, and enlargements of the boxed areas. Extracellular lactoferrin staining co-localizes with DAPI staining of DNA, indicating the staining represents NETs.
